# Whole-genome resequencing of Chinese indigenous sheep provides insight into the genetic basis underlying climate adaptation

**DOI:** 10.1186/s12711-024-00880-z

**Published:** 2024-04-02

**Authors:** Meilin Jin, Huihua Wang, Gang Liu, Jian Lu, Zehu Yuan, Taotao Li, Engming Liu, Zengkui Lu, Lixin Du, Caihong Wei

**Affiliations:** 1 Institute of Animal Sciences, Chinese Academy of Agricultural Sciences, Beijing, China; 2 National Animal Husbandry Service, National Center of Preservation and Utilization of Animal Genetic Resources, Beijing, China; 3 College of Animal Science and Technology, Yangzhou University, Yangzhou, China; 4 Lanzhou Institute of Husbandry and Pharmaceutical Sciences, Chinese Academy of Agricultural Sciences, Lan-Zhou, China

## Abstract

**Background:**

Chinese indigenous sheep are valuable resources with unique features and characteristics. They are distributed across regions with different climates in mainland China; however, few reports have analyzed the environmental adaptability of sheep based on their genome. We examined the variants and signatures of selection involved in adaptation to extreme humidity, altitude, and temperature conditions in 173 sheep genomes from 41 phenotypically and geographically representative Chinese indigenous sheep breeds to characterize the genetic basis underlying environmental adaptation in these populations.

**Results:**

Based on the analysis of population structure, we inferred that Chinese indigenous sheep are divided into four groups: Kazakh (KAZ), Mongolian (MON), Tibetan (TIB), and Yunnan (YUN). We also detected a set of candidate genes that are relevant to adaptation to extreme environmental conditions, such as drought-prone regions (*TBXT*, *TG*, and *HOXA1*), high-altitude regions (*DYSF*, *EPAS1*, *JAZF1*, *PDGFD*, and *NF1*) and warm-temperature regions (*TSHR*, *ABCD4*, and *TEX11*). Among all these candidate genes, eight *ABCD4*, *CNTN4*, *DOCK10*, *LOC105608545*, *LOC121816479*, *SEM3A*, *SVIL*, and *TSHR* overlap between extreme environmental conditions. The *TSHR* gene shows a strong signature for positive selection in the warm-temperature group and harbors a single nucleotide polymorphism (SNP) missense mutation located between positions 90,600,001 and 90,650,001 on chromosome 7, which leads to a change in the protein structure of TSHR and influences its stability.

**Conclusions:**

Analysis of the signatures of selection uncovered genes that are likely related to environmental adaptation and a SNP missense mutation in the *TSHR* gene that affects the protein structure and stability. It also provides information on the evolution of the phylogeographic structure of Chinese indigenous sheep populations. These results provide important genetic resources for future breeding studies and new perspectives on how animals can adapt to climate change.

**Supplementary Information:**

The online version contains supplementary material available at 10.1186/s12711-024-00880-z.

## Background

Extreme climate change can have a significant effect on livestock health, and thus reduce economic efficiency and animal welfare. Sheep (*Ovis aries*) are thought to be among the first domesticated livestock species [1], with the domestication process estimated to have originated in the Fertile Crescent approximately 11,000 years ago [2, 3]. Since their initial domestication, natural and artificial selection have driven sheep to remarkable phenotypic diversifications in appearance, growth, local adaptability, fertility, coat color, and meat palatability [4]. The global sheep population comprises more than 1000 breeds [5]. According to the national list of animal genetic resources for 2021, China has 89 domestic sheep breeds, including 44 indigenous breeds, accounting for nearly 4.4% of the world’s sheep breeds. China covers an extensive territory featuring a wide diversity of geographical, climatic, and ecological characteristics. China’s climate ranges are extremely large in terms of temperature (between -42 and 34 °C), humidity, and altitude, and these features created conditions that have promoted a high level of diversity and the evolution of a varied series of genetic adaptations in sheep. In particular, Chinese indigenous sheep have the ability to adapt to complex local environments, which makes them relevant model organisms for investigating genetic diversity and the mechanisms that underlie their environmental adaptation.

Understanding these genetic changes is becoming increasingly important with the rapidly changing global climate, because breeders aim at producing sheep with better climate tolerance. Extreme environmental pressures are widely perceived as significant drivers that shape the genome of animals [6]. Identifying potential molecular mechanisms that underlie climate adaptation driven by extreme environments has also become a key focus of evolutionary biology. Genetic variations influenced by environmental adaptation have been reported in various livestock species, such as pigs [6], chickens [7], sheep [8], goats [9] and cattle [10]. Current technology allows whole-genome sequencing on a large scale for the identification of signatures of selection related to important traits. Population genomics has been applied effectively and extensively to identify candidate genes associated with phenotypic diversity and biological, agricultural, and biomedical important traits in domestic animals. However, only a handful of such variants have been identified in sheep, which remain largely unexplored [8, 11, 12].

**Fig. 1:**
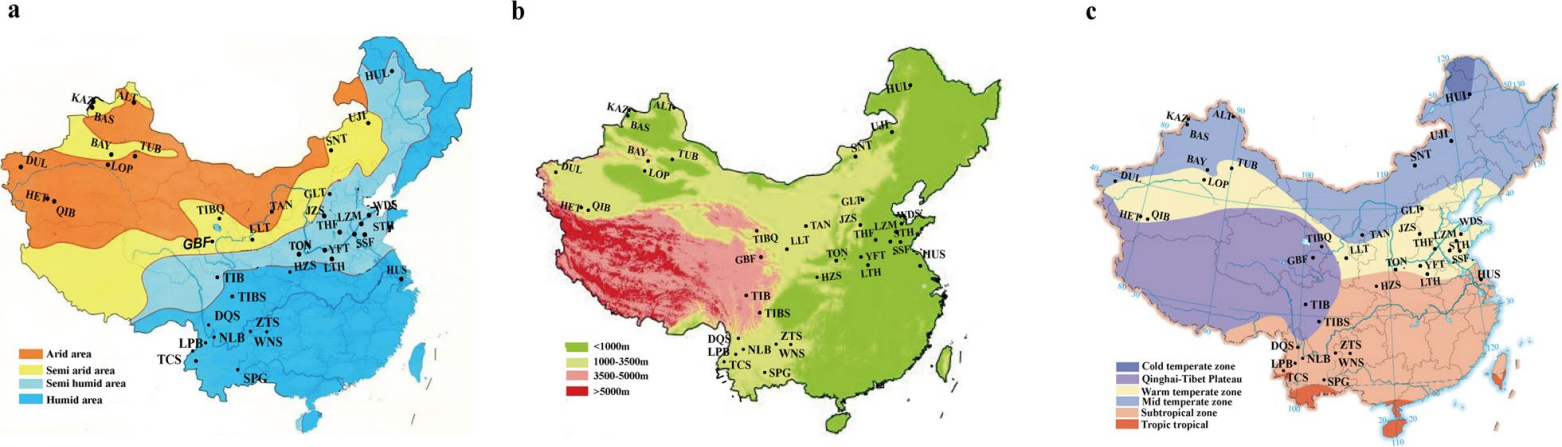
Geographic distribution of the sheep breeds included in this study. **a** Geographic variation of the annual mean precipitation (mm) and a geographic map indicating the distribution of the sheep breeds sampled for this study. **b** Sample information and geographic variation of the altitude in this study. **c** Sample information and geographic variation of the temperature in this study. The temperature band is divided according to the distribution of accumulated temperature

In this study, we conducted whole-genome re-sequencing of 173 individuals from 41 sheep breeds from various environments (Fig. 1) and (see Additional file 1: Table S1), with a mean genome coverage of 6.08x (from 5.46x to 13.14x) and we obtained 3800 Gb data in total. We performed a comprehensive analysis of the population structure of these sheep breeds and used the data to study genetic variants and genomic regions under selection and gain insight into novel molecular mechanisms of adaptive evolution in Chinese indigenous sheep.

## Methods

### Sample preparation and sequencing

A large panel of 173 sheep was selected for a comprehensive analysis of the population structure and of selective sweeps related to environmental adaptative changes, including 37 Chinese indigenous sheep breeds (n = 161) and four European sheep breeds (n = 12) (see Additional file 1: Table S1). The four European sheep breeds were set as the outgroup to analyze the population structure of Chinese indigenous sheep breeds. Genomic DNA was extracted from blood. For each individual, 1 to 3 µg of genomic DNA were sheared into fragments of 200 to 800 bp using the Covaries system. DNA fragments were then treated according to the Illumina DNA sample preparation protocol: fragments were end-repaired, A-tailed, ligated to paired-end adaptors and PCR amplified with 500-bp inserts for library construction. The DNA library was sequenced on the Illumina HiSeq 2000 platform using 100-bp paired-end reads. Each sample was sequenced on one or two lanes to achieve a mean coverage of 5.46x to 13.14x.

The Burrows-Wheeler Aligner (BWA v0.6.2) [13] was used to map paired-end reads to the sheep reference genome (ARS-UI_Ramb_v2.0). First, the reference was indexed. Second, the command ‘aln -o 1 -e 50 -I -i 10 -q 0 -t 2’ was used to find the suffix array coordinates of good matches for each read. Third, the command ‘sample -a 750’ converted suffix array coordinates to pseudo-chromosomal coordinates and paired reads to a BAM file. The other parameters were set to default values. The BAM file was then indexed and sorted according to genomic location using the Samtools 1.9 software [14]. Duplication reads were removed using sambamba markdup (v0.5.5) [15].

### Detection of variants and genotyping validation

We implemented the Genome Analysis Toolkit (GATK) v3.4 [16] variant quality score recalibration (VQSR) to get high-confidence variants. Considering that there are no sheep truth data in the GATK bundle, to generate truth and training datasets, we extracted the sites that were concordant between different SNP callers, including GATK v3.4 [16], Samtools 1.9 [14] and FreeBayes 1.3.1 [17], and a prior likelihood was set to Q10. The default settings of the GATK v3.4 VQSR were used to detect variants. SNPs that failed to pass quality controls were removed. Insertions/deletions (INDEL) with a missing rate higher than 10% were removed, and SNPs with a missing rate higher than 10% and more than two alternative alleles were also removed. Variants were annotated with predicted functional consequences using ANNOVAR [18]. The source databases used by ANNOVAR during annotation included the SNP database (dbSNP) Build 143. Before haplotype phasing, we excluded the SNPs that had a missing rate higher than 10% and more than two alternative alleles. Then, Beagle v5.4 [19] was used for imputation with the setting “window = 3000 overlap = 600”. Haplotype phasing was conducted using the SHAPEIT2 program [20].

To assess the accuracy of genotype calls from sequencing data, we performed genome-wide genotyping on six individuals using an Ovine 50 k SNP chip and achieved an overall non-reference discrepancy rate (NRD) of 0.99% on genotypes called from sequencing and genotyping (ranging from 0.89 to 1.07% per individual), which indicates that, in this study, the genotype calls from sequencing data were very accurate.

### Population structure analyses

We carried out analyses of population structure for all sheep breeds to determine the genetic clusters within the population. To reveal phylogenetic relationships, we constructed a neighbor-joining (NJ) tree using Fitch from the PHYLIP package based on the *F*_ST_ distance matrix, which was calculated using the EIG4.2 software [21]. *F*_ST_ was calculated using the Hudson estimator to evaluate population differentiation among the breeds. To eliminate the effect of admixture in the tree construction, we used the TreeMix 1.13 software to infer population splits and admixtures, allowing up to nine mixing events [22]. This method builds a bifurcating tree of populations and then identifies potential gene flow events based on the residual covariance matrix [23]. Genetic structure was inferred using the Admixture 1.3.0 software [24], which implements a block-relaxation algorithm. Default methods and settings were used in the admixture analysis. The EIG 4.2 software [21] was used to conduct a principal component analysis (PCA) on the autosomal biallelic SNPs. Eigenvectors from the covariance matrix were generated with the Eigen function using R version 3.6.3.

### Detection of selective sweeps during environmental adaptation

To detect positive selection during the environmental adaptation of Chinese indigenous sheep, we selected several representative Chinese indigenous sheep populations to perform a selective sweep analysis. We used two approaches based on the SNPs that had less than 10% missing data and a minor allele frequency higher than 5%. We calculated *F*_ST_ statistics and cross-population extended haplotype homozygosity (XP-EHH) scores [25] to detect selection signals across the whole genome. The top 5% of the *F*st values was determined to be the threshold for *F*_ST_ outliers, corresponding to the top 5% of the empirical distributions of all tested SNPs. Fisher’s exact tests were used to identify the 50,000-bp windows including significant SNPs and the enrichment significance threshold (*P* < *0.05*), which defined the candidate selection region. We calculated weighted *F*_ST_ values within consecutive, non-overlapping windows of 50,000 bp using the VCFTools v0.1.16 software [26]. Then, we drew a Manhattan plot for the weighed *F*_ST_ values and defined outliers as selective sweeps.

In addition, we calculated the XP-EHH score at each site with a minor allele frequency higher than 0.05 using the default settings in the selscan 1.0.3 software [27]. To compare genomic regions across populations, we divided the genome into consecutive, non-overlapping regions of 50,000 bp. For the XP-EHH selection scan, we assigned the maximum absolute value of the XP-EHH score in each window as the region statistic. We calculated the statistical significance for each 50,000-bp region from the empirical distribution of each test statistic. Windows with a significantly high statistic (the 1% right tail) of the empirical distribution were defined as selective sweeps. Adjacent sweeps within a distance of 50,000 bp were merged into one sweep.

Putatively selected regions were defined as genetic regions with overlapping SNPs that had extremely high *F*_ST_ values (top 5% level) and high XP-EHH values (top 5% level). We performed XP-EHH, Tajima’s D, and π ratio analyses to identify the top candidate region within the 1000-bp sliding window. XP-EHH was calculated using the selscan 1.0.3 software [27], and Tajima’s D and π ratios were estimated using a sliding window approach with the VCFTools v0.1.16 software [26].

### Gene ontology analysis

We used the US National Center for Biotechnology Information (NCBI) gene annotations to identify genes that were within candidate selective sweep regions. We tested for enrichment of gene ontology (GO) terms as assigned to the subset of these candidate genes. Orthology was established using the web application KOBAS 3.0 (http://kobas.cbi.pku.edu.cn/genelist/) [28]. The selected background organism was *Ovis aries.*

### Functional SNPs

To predict gene function, SNPs were annotated using the SnpEff v4.3 software [29]. The conservation scores among 12 species were calculated using PolyPhen-2 (http://genetics.bwh.harvard.edu/pph2/) for each missense mutation [30]. The Swiss model was used to predict the 3D protein structure of the relevant genes [31]. Missense3D was used to predict the structural changes introduced by an amino acid substitution (http://missense3d.bc.ic.ac.uk/missense3d/) [32].

## Results

### Characterization of the variants

Genome alignment indicated a mean of 98.1% sequencing coverage and 6.08-fold depth for each individual relative to the sheep reference genome [33] (see Additional file 2: Table S2). After the quality control procedures, we detected 46.6 million single nucleotide variants (SNVs), among which 5.94% were not found in the dbSNP build 143 (see Additional file 2: Table S2). Hence, our samples contributed substantially to the SNV database. Our results showed that 28.6% of the SNVs were located in genes (see Additional file 3: Table S3), among which 0.64% were in exons. After eliminating the effects of exon length, the mean number of SNVs in exons remained much smaller than that in the intergenic regions, which is consistent with the higher selection pressure on exons [34]. The ratio of non-synonymous to synonymous mutations was 0.75, again illustrating the higher selection pressure on coding regions. We identified 2035 SNVs in nonsense mutations that could terminate the expression of the respective genes (see Additional file 3: Table S3). Approximately one million INDEL were identified in our samples among which 30% were located in genes. Among the genic INDEL, 0.11% (3804) were in exons and ~ two-thirds of these led to frame-shift mutations and interrupted gene expression, as the 79 INDEL-induced stop-codon mutations. We also identified 21,876 structural variations, among which more than 45.58% overlapped with transposon elements (LTR/LINE/SINE) (see Additional file 4: Table S4).

### Population genetic characterization of sheep

**Fig. 2:**
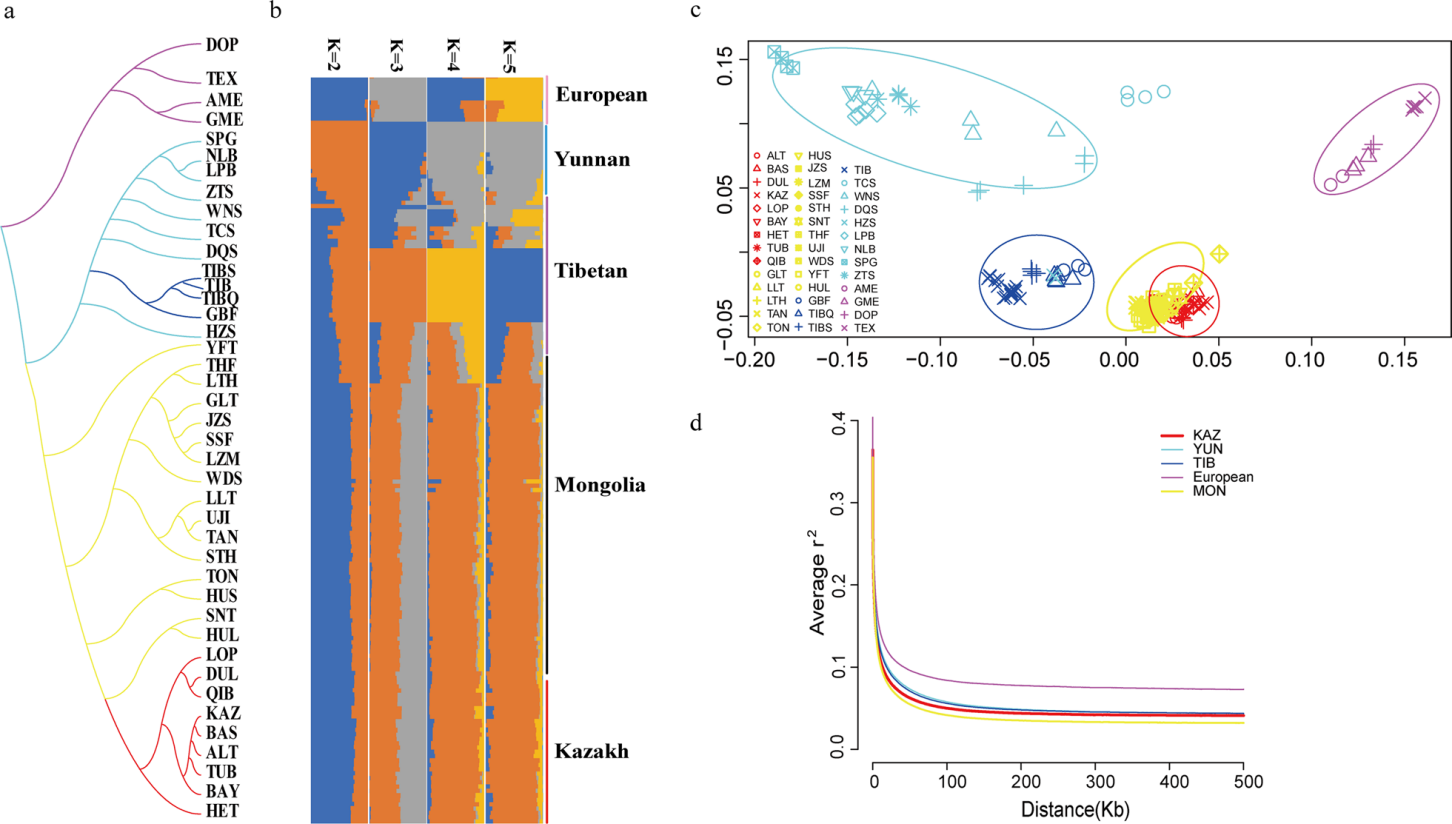
Current geographic populations of sheep and inferred genetic populations. In total, 173 sheep representing 41 Chinese indigenous sheep breeds were included in this study. Pink, blue, purple, yellow, and red dots indicate European, Yunnan, Tibetan, Mongolia, and Kazakh sheep groups. **a** Phylogenetic tree constructed by Fitch. **b** Population ancestry inferred by admixture analysis. **c** Fine-scale population structure based on the principal component analyses. **d** Decay of LD measured by r^2^ in the five groups

We performed a population structure analysis with the Admixture 1.3.0 algorithm [24]. The results showed that the four breeds in the Yunnan (YUN) group [Shiping Gray sheep (SPG), Ninglang Black sheep (NLB), Lanping Black-bone sheep (LPB), and Zhaotong sheep (ZTS)] were relatively pure, and all the other Chinese indigenous sheep breeds were mixtures. Figure 2b shows the results for K values ranging from 2 to 5. For K values larger than 2, a predominant component separated the YUN group from the others. For all K values, the Kazakh (KAZ) and Mongolian (MON) groups shared similar components with one dominant ancestral component. Only a few of the breeds of the Tibetan (TIB) group were intermediate between the YUN and KAZ/MON groups, but most of them formed a separate and clear cluster.

A phylogenetic tree was constructed to understand the population structure (Fig. 2a), and the European group was set as outgroup. The phylogenetic analysis also indicated that the MON and KAZ groups were close to each other, which is consistent with the PCA and population structure analysis (Fig. 2b and c). Linkage disequilibrium (LD) analysis revealed that LD decayed to half its maximum within less than 10,000-bp for European sheep breeds (Fig. 1d). Comparison of LD among the sheep populations showed that the European populations had a higher level of LD than the Asian populations. The KAZ and MON groups showed significantly different trends in LD decay, while for the YUN and TIB groups the trends were indistinguishable. The KAZ and MON groups showed a rapid decay rate and a low level of LD, while for the YUN and TIB groups the decay rate was slow and the level of LD was high. These results strongly suggest that Chinese indigenous sheep can be classified geographically into four groups: KAZ, MON, TIB, and YUN groups, and that Chinese indigenous sheep breeds may originate from the northern sheep population. Sheep that entered China were transported along the path Xinjiang-Inner Mongolia-Qinghai-Tibet Plateau-Yunnan-Guizhou Plateau for food and clothing [35], and then spread from drought to moist areas, from cold- to warm-temperature areas, and from low- to high-altitude areas.

### Selective sweeps for environmental adaptation

#### Screening for candidate genes associated with drought adaptation

The breeds included in our study had many biological characteristics that were associated with valuable genetic resources for exploring the genetic mechanisms of sheep adaptation. We selected sheep breeds that live near the Taklimakan desert, which is an arid and semi-arid area in China. Sheep breeds from semi-humid and humid areas were selected as a reference group to identify candidate genes related to drought adaptation (see Additional file 5: Table S5, see Additional file 6: Table S6, and see Additional file 7: Table S7).

**Fig. 3:**
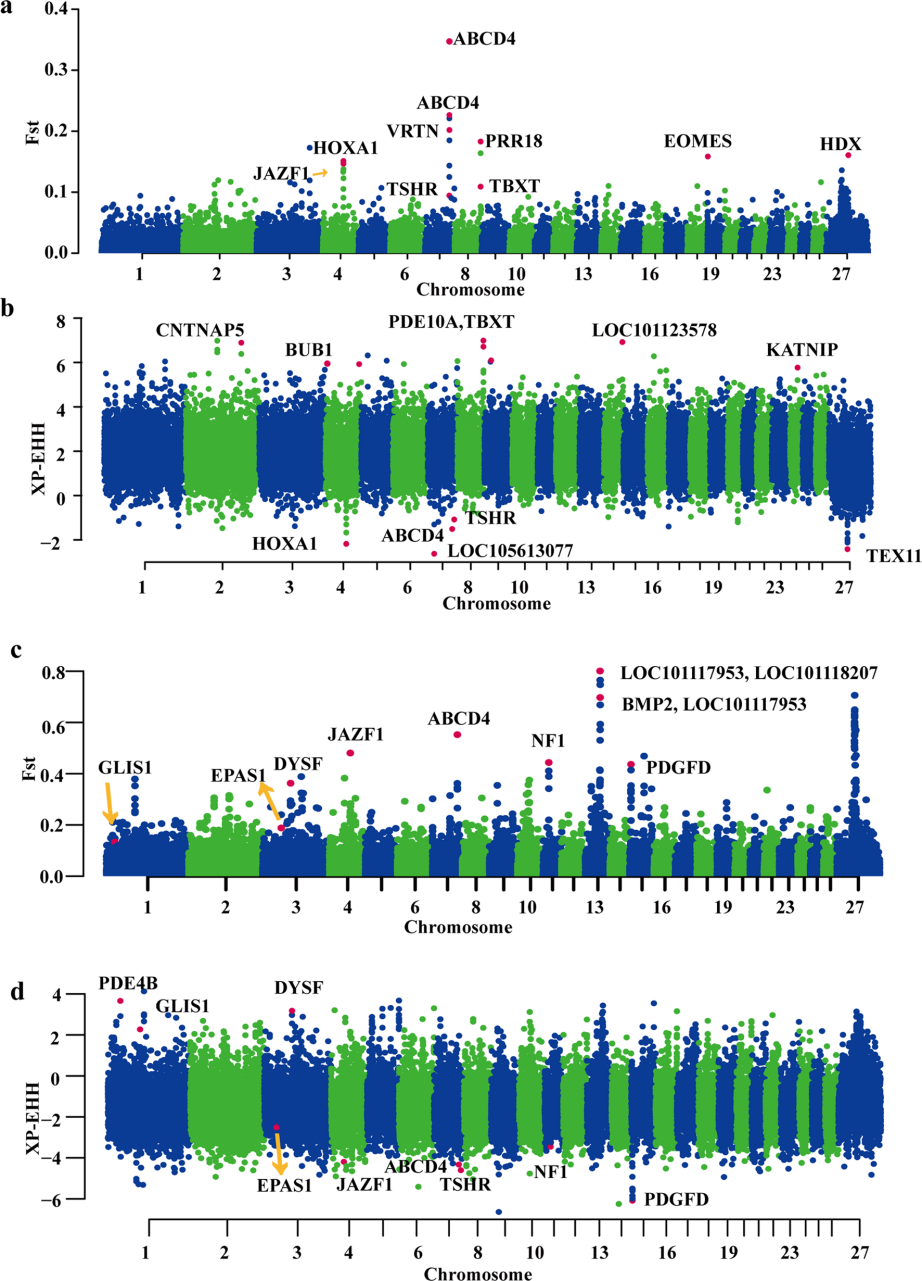
Positive selection scans for adaptation to drought and high-altitude conditions. *F*st and XP-EHH for 50-kb windows are plotted along the ovine whole genome. Chromosomes are represented by color. Genomic landscape of the *F*st values (**a**) and XP-EHH values (**b**) in the genome of sheep breeds from drought areas. Genomic landscape of the *F*st values (**c**) and XP-EHH values (**d**) in the genome of sheep breeds from high-altitude areas

In this study, we detected 876 candidate genes associated with drought adaptation (Fig. 3a and b). Among these candidate genes, seven are involved in the renin secretion pathway (*PLCB1*, *PLCB4*, *PDE1C*, *PDE3A*, *KCNMA1*, *PPP3CA*, and *PPP3CB*), nine in salivary secretion (*PR*, *PLCB1*, *PLCB4*, *ADCY1*, *CHRM3*, *ADCY8*, *KCNMA1*, *ATP1B1*, and *PRKG1*), and 11 (*PLCB1*, *PLCB4*, *ADCY1*, *CAMK2D*, *ADCY8*, *DSC1*, *PPP1R12A*, *PPP3CA*, *PLA2G4A*, *CACNG3*, and *PPP3CB*) in the oxytocin signaling pathway. These pathways are functionally related to the regulation of water retention and reabsorption in renal cells and blood vessels in the kidney. These genes were also enriched in the pancreatic secretion pathway and may be important for sheep adaptation to a desert environment. Several pathways related to estrus were enriched, such as the thyroid hormone signaling pathway, GnRH signaling pathway, and thyroid hormone synthesis. In addition, we detected six candidate genes that were enriched in the longevity-regulating pathway (*TP53*, *ADCY1*, *ADCY8*, *PPARGC1A*, *KL*, and *ATG5*). Among all these genes, the *PLCB1*, *PLCB4*, *PPP3CA*, *ATP1B1*, *PPP3CB*, and *ADCY8* genes were repeatedly enriched in drought-related pathways (see Additional file 8: Table S8).

We found significant GO terms for sheep breeds from the arid zone (see Additional file 8: Table S8), which were enriched for metabolic processes (GO:2000378, GO:0001523), bone development (GO:0060348), potassium channel activity, and sodium: bicarbonate symporter activity (GO:0005249), which indicate that body size, and energy metabolism play an important role in sheep adaptation to drought-prone environments. In fact, all these GO terms are biologically related to regulating water retention, and reabsorption in the body. These terms were explicitly related to sheep survival in an arid environment.

#### Screening for candidate genes associated with high-altitude adaptation

High-altitude sheep breeds near the Qinghai-Tibet Plateau were selected for the experimental group, and the low-altitude sheep breeds as the reference group (see Additional file 9: Table S9). Five hundred and sixty candidate genes were selected with the top 5% *F*_ST_ and XP-EHH values (Fig. 3c and d; and see Additional file 10: Table S10 and Additional file 11: Table S11). Regarding the genes related to high-altitude adaptation, the *TSHR*, *NPY*, and *GNAI1* genes were enriched in the lipolysis regulation part of the adipocyte pathway, *PDGFC*, *PDGFD*, and *NF1* in the EGFR tyrosine kinase inhibitor resistance pathway, *CREB5* and *DYNC1I2* in the vasopressin-regulated water reabsorption pathway, *TCF4*, *LEF1*, and *GNAI1* in the melanogenesis pathway, and *CARD10*, *CXCL12*, and *EDAR* in the NF-kappa B signaling pathway. We scanned the signatures of selection for high-altitude adaptation in Tibetan sheep breeds, and found that several pathways related to hypoxia, angiogenesis, and energy metabolism were enriched, such as melanogenesis, regulation of blood pressure, and negative regulation of reactive oxygen species metabolic processes.

In addition, the *EPAS1*, *NF1*, *JAZF1*, *MITF*, and *BMPER* genes were functionally involved in high-altitude adaptation, as previously reported [8, 11]. Notably, we observed a high *F*_ST_ value and a high XP-EHH value for the target gene *DYSF*, indicating a strongly selected region in this gene. *DYSF* is also a target gene of hypoxia-inducible factors (HIF), which stimulate the transcription of the *erythropoietin* gene during hypoxia at high altitudes [11]. Sharma et al. [36] have shown that the genetic disruption of the *DYSF* gene affects the expression of the vascular endothelial growth factor gene, thus Tibetan sheep under severe high-altitude stress may have a more effective energy metabolism. The *PDGFD* gene has a role in blood vessel development, and in high-altitude sheep breeds it has been shown to be a high-ranking candidate gene for the sheep fat-tail phenotype [37] and to regulate blood vessel development [38]. These findings indicate that these genes may play an important role in adaptive mechanisms in high-altitude environments.

We also found seven significant GO terms for Tibetan sheep (see Additional file 12: Table S12). These GO terms were associated with heart development (GO:0007507), pigmentation (GO:0043473), and glycerol metabolic processes (GO:0006071), which are relevant to high-altitude adaptation.

#### Screening for candidate genes associated with warm-temperature adaptation

**Fig. 4:**
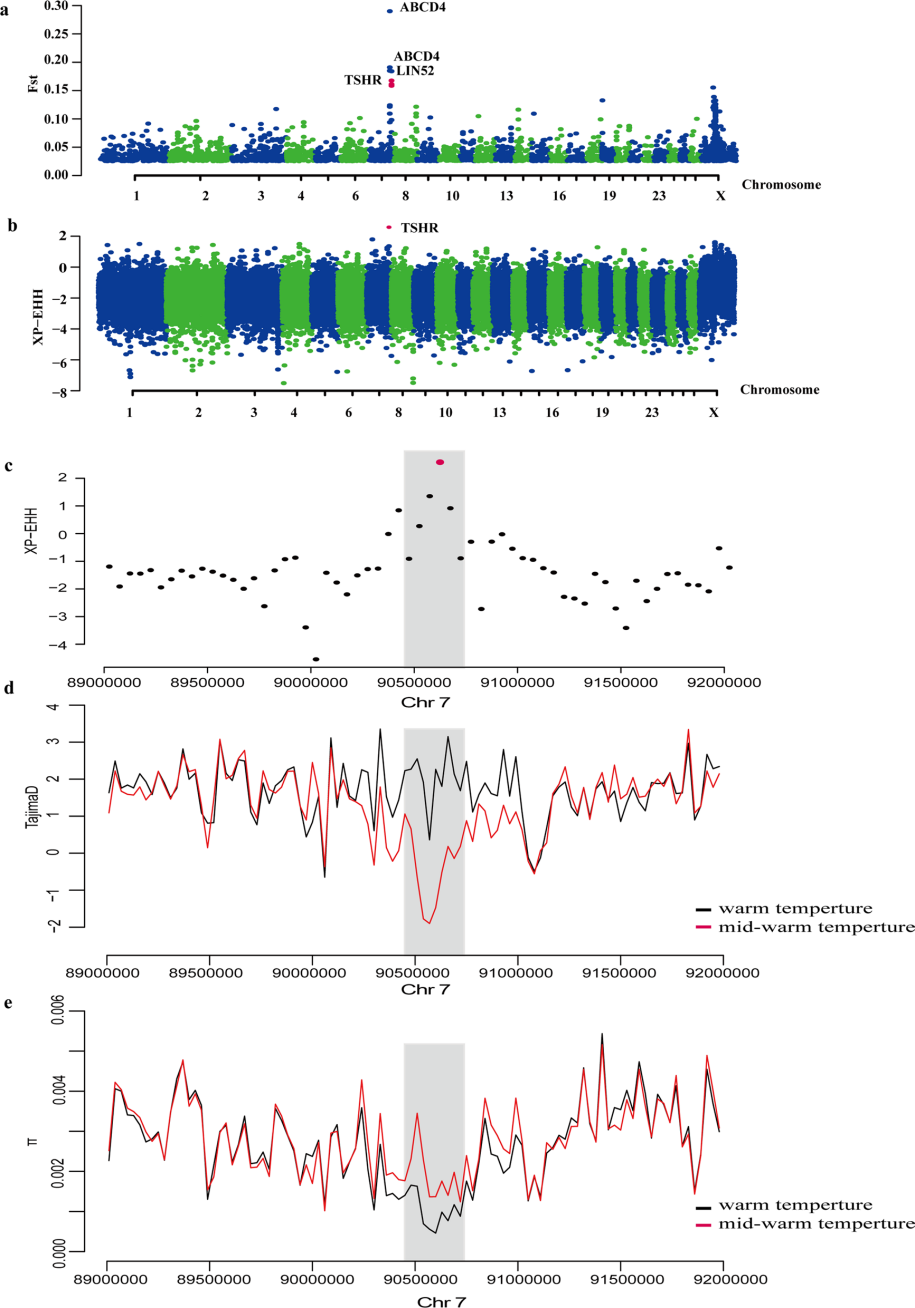
Positive selection scans for adaptation to warm-temperature areas. Sheep from warm-temperature areas are compared with those from mid-temperature areas. The population genetic differentiation *F*st values (**a**) and XP-EHH (**b**) within 50,000 bp sliding windows. The significant threshold for signatures of selection was set as the top 5% outliers for each test. The strongest positive signatures of selection are around the peak of the *TSHR* gene. The XP-EHH value (**c**), Tajima’s D (**d**) and πratio (**e**) are plotted against the peak position from 89 to 92 Mb on chromosome 7 within the 50,000-bp sliding window. The dark gray columns represent the strongest signatures of selection in this region

Next, we scanned sheep breeds living in warm environments for signatures of positive selection (see Additional file 13: Table S13). We calculated the *F*_ST_ and XP-EHH scores using the 50,000-bp sliding window approach (see Additional file 14: Table S14 and Additional file 15: Table S15) for sheep breeds from the warm-temperature area. Sheep breeds from mid-temperature areas were used as the reference group. The top 5% selection candidates that were common to all the tests were from the warm-temperature area, consistent with the separations of sheep populations from mid-temperature areas. Here, 324 candidate genes were identified in the warm-temperature group with the top 5% of *F*_ST_ values and XP-EHH values (Fig. 4a and b), among which, *PDGFD*, *ADCY1*, *PIK3CA*, *SKAP1*, *LPAR3*, *KITLG*, and *SIPA1L2* were enriched in the Rap1 signaling pathway, *TP53*, *ERBB4*, *TNR*, *PIK3CA*, *TLR2*, *JAK1*, *LPAR3*, *KITLG*, and *PDGFD* in the PI3K-Akt signaling pathway, *TSHR*, *PIK3CA*, *ADCY1*, and *PTGER3* in the lipolysis regulation of part of the adipocyte pathway and *PLCG2*, *TP53*, *PIK3CA*, and *DSC1* in the thyroid hormone signaling pathway. Moreover, the *ABCD4*, *SKAP2*, and *SLITRK2* genes were also selected in the warm-temperature group with *ABCD4* having a role in the number of vertebrae, which is an important economic trait in sheep production and increases body weight [39], *SKAP2* being involved in the immune system [40] and *SLITRK2* in the nervous system. Overall, we have genetic evidence for traits such as reproduction, adipose regulation, and metabolism that could be under selection during adaptation to warm areas.

Enrichment analysis for GO terms revealed significant terms for the warm-temperature group of sheep breeds (see Additional file 16: Table S16) that are enriched in metabolic processes (protein binding (GO:0005515), protein phosphatase binding (GO:0019903), protein phosphorylation (GO:0006468), ATP binding (GO:0005524), GTPase activator activity (GO:0005096)) and angiogenesis (positive regulation of vascular associated smooth muscle cell migration (GO:1904754), the epidermal growth factor receptor signaling pathway (GO:0007173), and platelet-derived growth factor receptor signaling pathway (GO:0048008). These terms are functionally related to heat loss regulation [41].

### Adaptation to extreme environments

Eight genes (*ABCD4*, *CNTN4*, *DOCK10*, *LOC105608545*, *LOC121816479*, *SEM3A*, *SVIL*, and *TSHR*) were identified in sheep adapted to drought-prone, high-altitude, and warm environments. We focused on the top positive selective sweep regions to minimize false positive signals in the warm-temperature group. The region with the strongest statistical signal included consecutive SNP positions and the *TSHR* gene, which was also identified in sheep adapted to drought-prone and high-altitude areas (Fig. 4a and b). Next, we refined the selection targets within the top candidate regions (*TSHR*) using three methods (XP-EHH, Tajima’s D, and π ratio) and we located a region on chromosome 7 between 89 and 92 Mb (Fig. 4c, d, and e). We detected two SNPs (SNP1: p.His271Pro and SNP2: p.Asp276Ala) within the most significant selected region on chromosome 7 between positions 90,600,001 and 90,650,001 bp that are missense variants in the *TSHR* gene (see Additional file 17: Table S17). They correspond to recent changes that may be particularly interesting for exploring gene functionality in sheep. The strong positive selection of *TSHR* may reflect the importance of seasonal reproduction in the adaptation of sheep to warm environments.

### Functional effects of the selected TSHR missense mutations

**Fig. 5:**
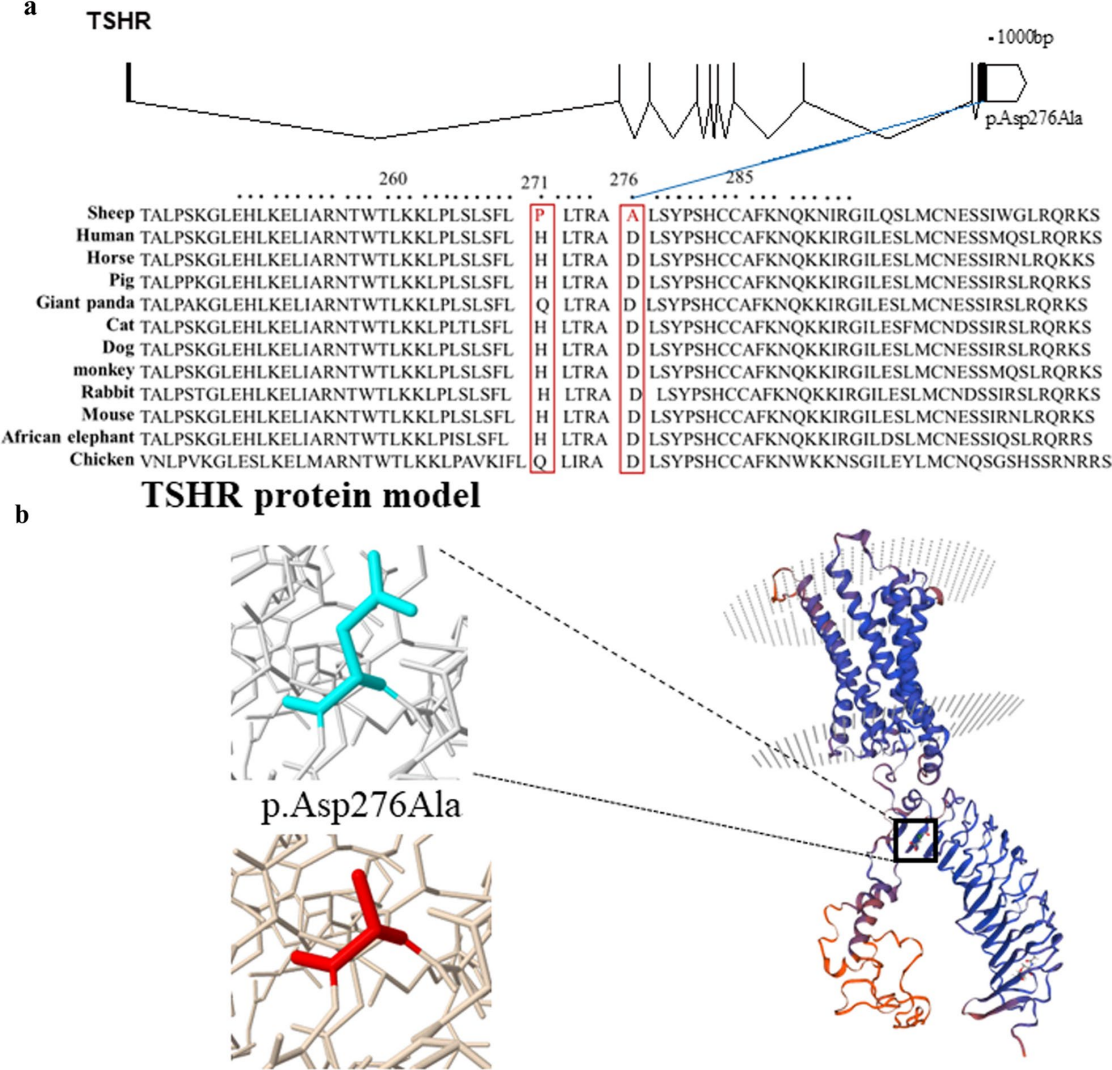
Annotation of the *TSHR* missense SNPs showing positive signatures of selection. **a** TSHR protein sequence analysis. The protein coordinates are based on the NP_001009410.1 NCBI protein. Protein sequences with the mutation present in sheep and 11 vertebrates are provided. **b** TSHR protein 3D structure model predicted by Swiss-model and Missense 3D. The left part of the panel shows the 276 amino acid change from Asp to Ala, with the wild type in turquoise and the mutant type in red

To further investigate the conservation of SNPs among other mammals, only the SNP2 (p.Asp276Ala) missense variant in the *TSHR* gene was found to be conserved in 12 different vertebrate species (Fig. 5a) and we investigated whether it has an impact on the protein structure. We used the Swiss-model software to predict the 3D protein model of THSR, and the missense 3D software to explore the 3D structure of TSHR with the amino acid change (Fig. 5b). After the substitution of the amino acid, the TSHR protein structure showed a buried H-bond breakage, an altered cavity, and a buried-to-exposed switch, which indicate that the protein structure and thus its stability were altered.

## Discussion

In this study, we sequenced the genomes of 173 sheep from 43 sheep breeds, of which 37 Chinese indigenous sheep breeds spanned a broad range of climates in terms of temperature, altitude, and humidity. In total, 46.64 million SNPs (3.60 million INDEL and SV) were identified based on our genome data. These data represent a valuable genetic resource for evolutionary analyses, particularly to help uncover functionally important variants that contribute to phenotypic diversity. In our study, the PCA and the NJ tree displayed indistinguishable results in the KAZ and MON groups, but the LD decay analysis showed that these two groups had different attenuation trends. We also observed that some breeds in the TIB group contained minor components from the western group, which could result from a common practice in the 1960s of raising the productivity of some Tibetan breeds through the introduction of Western breeds [42]. Our basic population genetic analysis revealed that Chinese indigenous sheep were divided into four groups (MON, KAZ, YUN, and TIB), which is not consistent with previous studies. Previous studies combined the KAZ and MON groups into a single population [8]. However, the patterns of LD strongly suggested a northern origin for the Chinese indigenous sheep, from which the Qinghai–Tibetan, and Yunnan–Kweichow breeds were subsequently derived [8], indicating a move from cold, drought-prone, and low-altitude areas to warm, humid, high-altitude areas. This result is consistent with the migration routes of eastern Eurasian sheep, which led from northern China to southern China, as deduced from a recent mitogenomic study [43]. These results strongly suggest that, geographically, Chinese indigenous sheep fall into four groups: KAZ, MON, TIB, and YUN, and that these breeds may originate from the northern population. Sheep were brought into China as resources for food and clothing, and transported via a route that led from Xinjiang to Inner Mongolia, then to Qinghai, the Tibetan Plateau, and Yunnan-Guizhou Plateau [35], and spread from cold, drought-prone, low-altitude areas to warm, wet and high-altitude areas.

### Adaptive mechanisms in drought-prone environments

Sweeping the sheep breed genome for positive signatures of selection for adaptation to drought-prone environments, we uncovered a series of genes that are involved in major signaling pathways related to the metabolism of glucose. Within the top candidate genes, *PCLB1*, *PCLB2*, and *CAMK2D* are functionally involved in the metabolism of arachidonic acid and inhibit K + transport (KEGG). The *ATP1B1*, *CACNG3*, and *KCNMA1* genes also influence ion transport to maintain homeostasis (KEGG). Animals that are too large to support their basal maintenance requirements under the resource limitations of drought-prone environments exhibit this phenomenon, which is also common in snakes that are adapted to extreme environments [44]. The *TBXT* gene regulates the number of caudal vertebrae [38] and body size in sheep [37, 45]. Therefore, it is reasonable to assume that *TBXT* is important for adaptation to drought-prone environments. Several candidate genes are in reproduction-related pathways (the GnRH signaling pathway and thyroid hormone synthesis pathway) [9]. One of the ways animals adapt to their environment and improve their survival rate is to actively reduce reproduction during dry periods [46]. Several candidate genes were also enriched in pathways related to the development of the kidney, which mainly excretes metabolites and regulates water, electrolytes, and acid–base balance [47]. The oxytocin signaling pathway was also enriched and is associated with water retention and reabsorption in blood vessels in the kidney [8]. Because of water and food scarcity in extreme environments, these pathways are critical for sheep survival. These pathways and candidate genes have also been identified in chickens [7], cattle [10], and goats [9], which indicates that these different species may have similar signatures of evolutionary adaptive mechanisms in response to drought-prone environments.

### Adaptive mechanisms in high-altitude environments

Tibetan sheep, as an indigenous breed in the high-altitude regions of Tibet (> 4000 m above sea level), has adapted to high-altitude hypoxic environments [11]. The enriched pathways detected in these sheep were related to hypoxic adaptation, angiogenesis, and energy metabolism and thus are functionally important for the adaptation of sheep to high-altitudes. Among the genes identified as likely targets of selective pressures, *EPAS1* has been consistently identified. The same selection targets have been described in a number of other organisms, including Tibetan horses [48], Tibetan dogs [49], yaks [50], and Sherpa (humans) [51], which indicates similar physiological and metabolic responses to hypoxic environments. High-altitude adaptation may be caused by multiple genes that act in concert with one another [52]. In addition, the target gene, *DYSF*, has a higher *F*_ST_ and XP-EHH value in the high-altitude group, which is also consistent with our previous study [11]. It has been shown that the *MITF* gene is amplified in certain melanoma [53] and thus it may be related to adaptation to high ultraviolet environments at high altitudes [11]. The *JAZF1* and *NF1* genes have also been associated with high-altitude adaptation by promoting angiogenesis [54, 55]. The *IGF2BP2* gene plays an important role in cell proliferation, epithelial-mesenchymal transition, and metabolism [56]. *IGF2BP2* knockdown protects human bronchial epithelial cells from hypoxia/reoxygenation injury by inactivating p38 MAPK, AKT, ERK1/2, and NF-кB pathways [57]. These results show that most of the genes located within the significant sweep regions detected in our study are functionally candidate genes underlying these domestication events. Distinct domestication events, adaptation to different altitude zones, and divergent selection in sheep have shaped their genomic differences [58]. Some of the genes identified in this study are promising candidates for further investigation to unravel the genetic control of traits associated with indigenous sheep breeds and represent potential relevant regulatory factors for high-altitude species to respond to hypoxic environments [52].

### Adaptive mechanisms in warm environments

Climatic conditions influence many aspects of an animal’s life, including reproduction and therefore can have important effects on livestock production systems [59]. By sweeping the genomes of sheep breeds that live in warm environments, for positive signatures of selection, we uncovered a series of genes that were related to energy metabolism and played important roles in regulating cellular responses to warm environments. Among these candidate genes, *SOX6* suppresses mitochondrial oxidative capacity and exercise performance, and *BMP2* triggers the BMP/SMAD signaling pathway, promoting both hyperplasia and hypertrophy of bovine preadipocytes [60]. We also uncovered some major signaling pathways associated with reproduction. Previous studies have demonstrated that short- and long-term exposure to heat stress significantly decreases semen production and quality of rams and impairs the fertility and fecundity of ewes, resulting in fewer lambs born per ewe mated [61]. It is worth noting that the *TSHR* gene was within the most strongly selected signature in the warm-temperature group. The same selection targets have been described in chicken [62]. Previous studies have established a pivotal role for *TSHR* in metabolic regulation and photoperiod control during reproduction in animals, such as sheep [63], herrings [64], geese [65], chickens [66], and rabbits [67]. In addition, an SNP in the *TSHR* gene resulted in an aspartic acid-to-alanine substitution, and it was conserved among most of the other species. We also hypothesize that this mutation causes structural protein damage. We speculate that temperature influences reproduction and that the *TSHR* gene may be involved in an increase in metabolic activity and growth. This result also provides additional evidence that temperature has large effects on most aspects of the reproductive function in mammals, including disruptions in spermatogenesis and oocyte development, oocyte maturation, early embryonic development, fetal and placental growth, and lactation [68]. At the protein level, our work reveals that a missense mutation in the *TSHR* gene may be important for adaptation to warm environments in sheep.

## Conclusions

Our study provides a comprehensive analysis of environmental adaptation in Chinese indigenous sheep breeds using whole-genome sequencing. It includes 37 breeds spanning different environments across China and reveals several novel genes, important pathways, and GO terms associated with local adaptation of sheep to plateau, desert, and warm environments. Specifically, *TSHR* was identified as the top candidate gene for adaptation to life in warm areas. Functional assays demonstrated that a missense mutation influences the stability of the TSHR protein. Thus, our findings provide new opportunities to the breeding industry in the form of novel selection targets for improving and facilitating breeding strategies, leading to a cost-effective sheep industry.

### Supplementary Information

**Additional file 1: Table S1.** a: sample information and b: weather information of regions where each breed lives.

**Additional file 2: Table S2.** Information on the variants for each breed.

**Additional file 3: Table S3.** Distribution of the variants in the sheep genome.

**Additional file 4: Table S4.** Statistics of structural variants overlapping with annotated transposon elements.

**Additional file 5: Table S5.** Sample information selected by drought and moist groups.

**Additional file 6: Table S6.** Top 5% selective sweep regions based on *F*st for sheep breeds from drought areas.

**Additional file 7: Table S7.** Top 5% selective sweep regions based on XP-EHH for sheep breeds from drought areas.

**Additional file 8: Table S8.** KEGG pathway and GO terms that show overlapping genes for the sheep breeds from the drought areas.

**Additional file 9: Table S9.** Sample information selected by high-altitude group and low-altitude group.

**Additional file 10: Table S10.** Top 5% selective sweep regions based on *F*st for sheep breeds from high-altitude areas.

**Additional file 11: Table S11.** Top 5% selective sweep regions based on XP-EHH for sheep breeds from high-altitude areas.

**Additional file 12: Table S12.** KEGG pathway and GO terms that show overlapping genes for the sheep breeds from the high-altitude areas.

**Additional file 13: Table S13.** Information of sample selected by warm-temperate and mid-temperature zone sheep breeds.

**Additional file 14: Table S14.** Top 5% selective sweep regions based on *F*st for sheep breeds from warm-temperature areas.

**Additional file 15: Table S15.** Top 5% selective sweep regions based on XP-EHH for sheep breeds from warm-temperature areas.

**Additional file 16: Table S16.** KEGG pathway and GO terms that show overlapping genes for sheep breeds from warm-temperature areas.

**Additional file 17: Table S17.** Amino acid changes in the TSHR protein.

## Data Availability

Sequencing and variants data are available via the National Center for Biotechnology Information database (BioProject ID: PRJNA521847 and PRJNA309636).
